# The Rise and Fall of HIV in High-Prevalence Countries: A Challenge for Mathematical Modeling

**DOI:** 10.1371/journal.pcbi.1003459

**Published:** 2014-03-13

**Authors:** Nico J. D. Nagelkerke, Paul Arora, Prabhat Jha, Brian Williams, Lyle McKinnon, Sake J. de Vlas

**Affiliations:** 1 Institute of Public Health, College of Medicine and Health Science, United Arab Emirates University, Al Ain, United Arab Emirates; 2 Department of Public Health, Erasmus MC, University Medical Center Rotterdam, Rotterdam, Netherlands; 3 Department of Medical Microbiology and Infectious Diseases, University of Manitoba, Winnipeg, Canada; 4 Center for Global Health Research, St. Michael's Hospital, Dalla Lana School of Public Health, University of Toronto, Toronto, Canada; 5 South African Centre for Epidemiological Modelling and Analysis, University of Stellenbosch, Stellenbosch, South Africa; 6 Department of Medicine, University of Toronto, Toronto, Canada; 7 Department of Medical Microbiology, University of Nairobi, Nairobi, Kenya; Imperial College London, United Kingdom

## Abstract

Several countries with generalized, high-prevalence HIV epidemics, mostly in sub-Saharan Africa, have experienced rapid declines in transmission. These HIV epidemics, often with rapid onsets, have generally been attributed to a combination of factors related to high-risk sexual behavior. The subsequent declines in these countries began prior to widespread therapy or implementation of any other major biomedical prevention. This change has been construed as evidence of behavior change, often on the basis of mathematical models, but direct evidence for behavior changes that would explain these declines is limited. Here, we look at the structure of current models and argue that the common “fixed risk per sexual contact" assumption favors the conclusion of substantial behavior changes. We argue that this assumption ignores reported non-linearities between exposure and risk. Taking this into account, we propose that some of the decline in HIV transmission may be part of the natural dynamics of the epidemic, and that several factors that have traditionally been ignored by modelers for lack of precise quantitative estimates may well hold the key to understanding epidemiologic trends.

## Introduction

**Figure 1:**
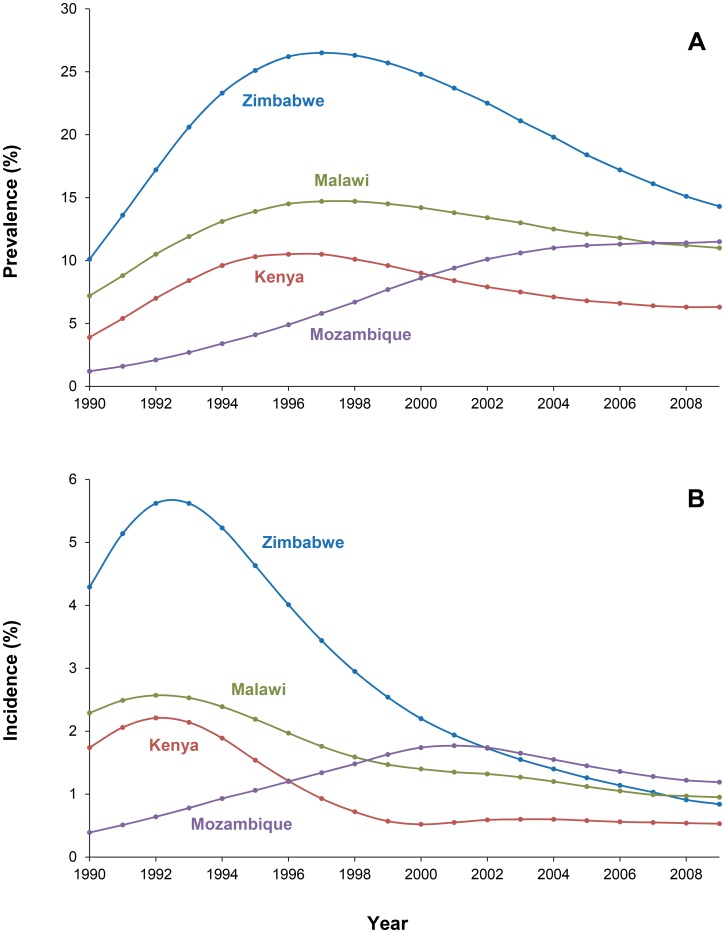
Adult (15–49) HIV prevalence (A) and annual incidence (B) in Zimbabwe, Kenya, Malawi, and Mozambique, 1990–2009 [8].

Since the discovery of the first HIV/AIDS case more than three decades ago, enormous progress has been made in understanding, treating, and preventing the infection. For example, there is now solid evidence that antiretroviral therapy (ART) taken by either HIV-infected or uninfected individuals can prevent HIV transmission [1]–[4]. Nevertheless, the epidemic keeps surprising us. In particular, it has been noted that in many countries, notably in highly affected sub-Saharan Africa, but also in India, HIV prevalence is dropping fast [5]. In Zimbabwe, once one of the worst affected countries, HIV prevalence levels have halved over the past 12 years, dropping from 30% in 1998 to 15% in 2011 [6]. However, Zimbabwe is certainly not the first country to experience such an epidemiological change, as in east and central Africa, declines have been observed as early as the mid-1990s (Figure 1A) [7]–[9].

While these drops in HIV prevalence are dramatic, underlying changes in HIV incidence must have occurred several years earlier and more rapidly (Figure 1B). This is because with an average survival time of approximately ten years (in the absence of treatment), even a sudden complete interruption in transmission would translate into a gradual decline in prevalence. In Nairobi, changes in infection risk seemed to occur in high-risk populations first, while HIV prevalence in the general population was still stable or increasing. In 1986, over 80% of sex workers were HIV-infected, but this declined to less than 50% after 1997. Also, incidence dropped dramatically in these sex workers [10]. In these women, the per–unprotected-sex-act rate of HIV acquisition fell over 4-fold between 1985 and 2005, and in a recent analysis of 2008–2011, HIV incidence was 2.2% in over 1,500 person-years of follow-up (McKinnon et al., submitted).

This rapid decline has occurred at a time when we still do not fully understand why HIV struck sub-Saharan Africa so exceptionally hard in the first place. At its peak, HIV prevalence in several countries in Africa exceeded more than 20% of the adult population, although there was a lot of heterogeneity both within and between countries [11]. The epidemic onset in sub-Saharan Africa was also often extremely rapid, with estimated prevalence doubling rates of as little as one year, implying that each HIV-infected person annually infects, directly or indirectly, another (susceptible) individual [12]. With transmissions apparently occurring over the entire infected period [13], this would seem to suggest high levels of the basic reproduction number *R*_0_ (i.e., the average number of secondary cases generated by an infected individual, over the duration of his infection, when all contacts are uninfected). This, however, seems to conflict with what studies have shown about HIV transmission (in)efficiency. For example, some 50% of HIV-affected couples are still discordant when discovered, and during follow-up the annual seroconversion rate of the HIV-negative partner has ranged from 2% to 12% [14]–[16]. With an average—untreated—survival after HIV infection being approximately ten years, this would seem to suggest that, in the heterosexual population, *R*_0_ cannot be much larger than one. With such a low *R*_0_, generalized heterosexual epidemics either cannot occur or only spread slowly [17]. This is, in fact, also what happened outside of sub-Saharan Africa.

This discrepancy between high-prevalence sub-Saharan Africa countries and the rest of the world has sparked many theories and debates about what made Africa “special." Indeed, there is still no full consensus about the reasons why various African countries experienced severe generalized HIV epidemics while most of the rest of the world did not. Most of the hypotheses focus on high-risk sexual behavior such as rapid partner change and low condom use, aggravated by transmission co-factors, notably other sexually transmitted infections that are spread by the same high-risk sexual behavior. Undoubtedly, highly connected sexual networks generated by unprotected, often transactional, sex with many different, frequently concurrent, partners are common in Africa [18]–[27]. This is also shown by the high levels of conventional, bacterial sexually transmitted infections (STIs) as well as HSV-2 in sub-Saharan Africa, infections that have been implicated as transmission co-factors, although their exact role is still moot [28]–[34]. These risk factors have been incorporated in many mathematical models of the HIV epidemic, models that have also been used to interpret the recent decline in HIV transmission. Several modeling studies interpreted the fall in HIV transmission, therefore, as a decline in the very factors that made sub-Saharan Africa special.

However, as throughout history epidemics have always come and gone, even without interventions, we should consider the possibility that the decline in HIV transmission may also have “natural" causes. Here we discuss findings, both epidemiological and from the basic sciences, which are currently ignored in HIV transmission models and suggest that some of the dramatic decline in HIV incidence may be part of the natural dynamics of the HIV epidemic and might have occurred even in the absence of any major behavior change. To fully explore the impact of these findings, they should be incorporated in future modeling studies.

## Current HIV Models, Common Assumptions, and Their Limitations

Mathematical modeling of infectious diseases is a highly developed science or technology, and software to accelerate the development and implementation of models is increasingly available [35]–[39]. For modeling complex situations that are difficult to capture in finite sets of (differential) equations, individual- or “agent-" based micro-simulation, such as used in STDSIM and SimulAIDS, is now well developed [40]–[42]. Advanced methods for fitting models to data and to incorporate uncertainty into inference and predictions have been used [43], [44]. From the very beginning of the HIV epidemic, mathematical modelers, together with epidemiologists and statisticians, have been heavily involved in shaping our understanding of the infection and its transmission [45]. They have also been instrumental in the interpretation of observed trends, in planning of prevention strategies, in guiding data collection, and in developing scenarios for the future course of the epidemic [46]–[48].

Most models, irrespective of their implementation and mathematical detail, incorporate a constant per-sexual-contact transmission risk, which may depend on the phase of infection of infected partners, and STI cofactor effects that increase this risk. Given these parameter values, infection can spread through dynamic networks modeled with variable complexity. This constant risk assumption implies that “pre-emptive saturation" is the only density-dependent mechanism at play [49]. That is, transmission will continue to increase until so many sex partners of HIV-infected individuals are already infected that (on average) each HIV-positive individual only infects one still uninfected individual. The latter condition defines endemic equilibrium, which will set in and remain “forever" unless behavior change or interventions shift this equilibrium to lower levels. Moreover, in homogeneous populations this equilibrium will be reached after a continuously increasing prevalence with perhaps a minor “overshoot," as mortality lags—by about a decade—behind incidence [50]. Thus, when incidence declines, the common fixed risk per sexual contact assumption implies the conclusion of substantial sexual behavior changes.

As in various countries the decline in HIV transmission started prior to the year 2000, major biomedical interventions such as antiretroviral therapy (ART) or male circumcision campaigns cannot explain the decline in HIV transmission. Most current models thus necessarily interpret the epidemiological change as evidence of (large) changes in sexual behavior or depletion (by higher AIDS mortality rates) of the group with the highest rates of partner change or as proof of success of behavioral interventions [51]–[54]. Several modeling studies have indeed come to this conclusion, although it is unclear whether direct empirical studies, mostly self-reported and therefore potentially biased, support commensurate declines in risk behavior [55]–[69]. While such behavior changes undoubtedly contributed to the decline in transmission [70], they may not be the only explanation. That other factors may play a role is supported by the observation that population behavior change appears to fail to explain the early, rapid declines in risk per unprotected contact among Kenyan sex workers [10].

## Non-linearity of the Relationship between the Number of HIV Exposures and Infection Risk

It is widely believed that HIV transmission is very inefficient. On average, a very small proportion of sexual encounters between infected and uninfected partners lead to transmission [71]. When transmission does take place, infection is often the result of a clonal expansion of a single founder virus, and the probability of any one virion (virus particle) successfully infecting a person must be on the order of one in a billion [72]. This is corroborated by SIV (monkey HIV) infection experiments, which often show a single cluster of infected cells in the female genital tract in the first few days of SIV infection [73]. Special factors, such as an STI cofactor effect, therefore have to be invoked to plausibly explain and model the observed epidemics.

Potentially misleading, however, may be the concept of per-contact transmission risk. Several observations disprove a constant per-coitus risk of transmission [74], [75]. Downs and Vincenzi, among the first authors to study this, have shown the relationship between the number of sexual contacts and risk of HIV transmission to be highly non-linear. In their study involving 563 heterosexual partners of HIV-infected subjects, the risk of transmission was 10% for those with less than ten unprotected contacts and increased to only 23% after 2,000 unprotected contacts [76], [77]. Thus, the first few sexual exposures are clearly more dangerous than subsequent ones. Sex worker contacts of Thai military conscripts were also associated with high per-contact risk, as were contacts among adult film actors [78]–[80]. Low-dose mucosal SIV challenge experiments in non-human primates suggest a similar phenomenon; while some monkeys become infected quite rapidly, others require far more challenges, at times with higher virus doses, to achieve infection [81]. It is unclear, however, how to unambiguously interpret this non-linearity with major implications for modeling and model predictions. Interestingly, another transmission non-linearity exists in the relationship between viral load and the risk of infection at each sexual contact. Each ^10^log increase in plasma HIV-1 RNA increases the per-act risk of transmission by 2.9-fold; that is, the transmission risk seems to be approximately proportional to the square root of the viral load [82]. A similar non-linear relationship appears to exist between HIV-1 RNA in the genital tract and the risk of transmission to an uninfected partner [83].

Perhaps, if (cumulative) risk is determined by numbers of virions to which a person is exposed, irrespective of the number of coital acts, then the non-linearity between number of contacts and transmission risk may well reflect the same biological phenomenon as between per-coitus viral dose and transmission risk.

## Biological Mechanisms of the Non-linearity in HIV Transmission

Below we present some mechanisms that may account for the non-linearity in the relationship between exposure and transmission risk.

### Susceptibility of the HIV-negative partner

As with everything biological, susceptibility to infection is likely to vary both among and within individuals. Early in the HIV epidemic, it was discovered that individuals homozygous for the Δ32 mutation of the CCR5 gene were practically resistant to HIV infection [84]. Other genetic factors also appear to play a role in susceptibility and resistance, but much of the genetics and biology of resistance have remained elusive, and behavior and acquired factors may also be important [85]–[89]. This raises the issue of whether the increased transmission during acute infection is totally due to an increased infectivity of the infected partner. Acute infection in the HIV-positive partner is often the first exposure of the negative partner to HIV, and it seems logical that the most susceptible individuals get infected first and fast.

**Figure 2:**
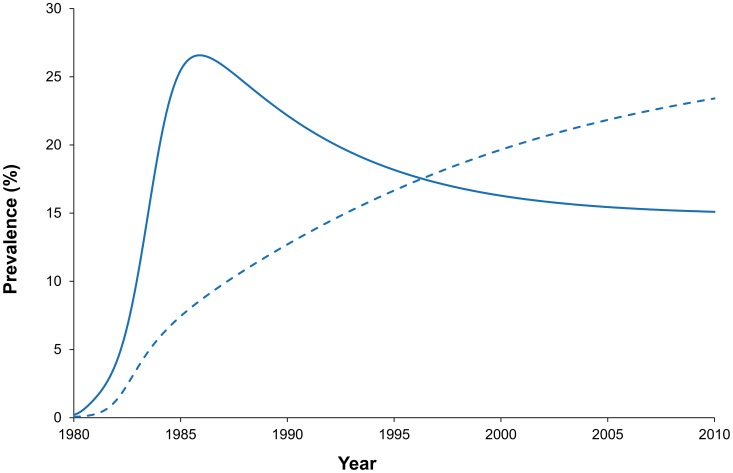
The projected course of the HIV epidemic (prevalence) under simplified assumptions about heterogeneity in susceptibility to HIV infection [50]. For comparison, the dashed curve shows the projected course in the absence of such heterogeneity, but with similar final size of the epidemic.

In statistical terms, when such heterogeneity is taken into account, the duration to infection (in terms of exposure) is a mixture of exponential distributions, with characteristically declining hazard rates; i.e., risk appears to decline with the total number of previous exposures [90]. As people can seroconvert only once, heterogeneity in susceptibility is notoriously hard to measure directly, but studies are highly suggestive that it can be substantial. Sex workers in Nairobi in the 1980s reached very high (>80%) infection levels, but the risk of seroconversion was strongly negatively associated with duration of exposure and there was even evidence of a small, highly resistant subgroup [91]. In our most recent analysis, this decrease in risk was 24% per year of exposure (McKinnon et al., submitted). The same has been observed in other sex worker cohorts [92]. Whether heterogeneity in susceptibility between individuals offers a totally satisfactory explanation of the rapid HIV epidemiological change is still unclear. By itself, it seems to fail to satisfactorily explain the onset of the epidemic. It may be of interest that for the same final size of the epidemic, heterogeneity in susceptibility could give rise to faster epidemic onset. Perhaps because of the difficulty of obtaining empirical estimates of the level of heterogeneity in susceptibility, this variable has usually been ignored in HIV epidemic models. Yet, heterogeneity in susceptibility and resistance can have a major effect on the course of the epidemic. In fact, models that incorporate this heterogeneity predict that prevalence peaks and then drops, as actually observed (Figure 2) [50]. Heterogeneity in susceptibility in its most extreme form, where only a subset of individuals are (highly) susceptible, and the rest minimally susceptible, may also lead to a fragmentation of sexual networks as all connections (edges) via non-susceptibles (nodes) are effectively removed. Thus, sexual networks consisting of loosely connected “cliques" would fragment into disjointed sub-networks, with obvious implications for HIV spread. Heterogeneity in susceptibility over time, within individuals, almost certainly also exists but, except in the context of STIs, has remained largely elusive.

### Immunity

Current mathematical models of HIV ignore the possibility of naturally acquired immunity. There is evidence of (cellular and other) immune responses to HIV among exposed but uninfected individuals, some of which are associated with reduced infection risk, but whether such responses are evidence of (long-term) protective acquired and/or adaptive immunity is unclear [93]–[97]. The success of the RV144 vaccine trial in Thailand should also alert us to the possible existence of forms of protective immunity to HIV [98], [99]. Immunity could develop, for example, after exposure to defective virions, i.e., virus particles unable to establish a reproductive infection. Given these data, the non-linearity of the relationship between number of contacts and transmission risk may also be due to such acquired immunity, including mucosal immune mechanisms such as HIV target cell availability, for example [100]. If so, this immunity perhaps may only offer protection against one of several strains, or subtypes (clades) of the virus, with implications for the risks of partner change [101].

Another form of immunity that may be relevant is alloimmunity, i.e., immunity to (HLA) antigens expressed by sex partners. Early studies demonstrated potent anti-HIV activity induced by allo-immunization in humans [102]. There is evidence that transmission often takes place by HIV hitching a ride in cells. As such cells express HLA molecules of the host, alloimmune responses may be protective [103]. Evidence, albeit somewhat conflicting, for the importance of these responses comes from both mother-to-child and sexual transmission studies [104]–[108].

With the high strain diversity of HIV and the extremely high degree of polymorphism of HLA antigens, one would expect regular partners of HIV-infected individuals to benefit much more from these types of immunity than (say) sex workers whose immune systems have to cope with widely divergent HIV strains and HLA antigens and whose alloimmune responses may even be compromised by their high levels of concurrency [109]. If the non-linearity of risk is due to some form of specific immunity, then this would imply much greater risks associated with multiple and/or concurrent partnerships than if it were due to, for example, innate, broadly protective, susceptibility factors. New HIV-positive partners would entail a very high risk during the first couple of contacts, whereas innate resistance or susceptibility would not discriminate between contacts with the same and with different HIV-infected partners.

Interestingly, with alloimmunity, the risk associated with acute phase infection of a seroconverting regular partner would be much lower than the risk associated with a new seroconverting partner, as it (the alloimmunity) is acquired *before* HIV exposure. This would not be the case with immunity against the virus itself. It seems likely that this may affect the relative importance of concurrent versus new partnerships. To our knowledge, no study has explored the implications of these types of immunity for the spread of HIV. Infections for which immunity is a density-dependent mechanism, such as measles, are often characterized by epidemiological patterns of (periodic) violent outbreaks, followed by subsequent declines when high levels of immunity in the population (herd immunity) form an impediment to ongoing transmission. Therefore, the epidemiological implications of HIV immunity merit further study.

### Heterogeneity in infectiousness

As viral load varies among people, one can expect some individuals to be greater shedders of infectious virions, and thereby more infectious, than others. Early-stage, acute infection has been associated with high viral loads and high infectiousness, and this seems to be the preferred “consensus" interpretation of the non-linearity of the relationship between exposure and risk. This acute infection period (first few months) has been estimated to be up to 26 times as infectious as the subsequent stage of infection [13], [110]. Yet, paradoxically, the elevation in transmissibility observed during acute infection appears to be greater than would be expected based on viral load alone (although not all virions may be equally infectious), and direct evidence implicating acute infections only comes from a single study that ignored alternative explanations and whose methodology has been questioned [111]–[113]. Nevertheless, several mathematical models have incorporated this aspect of transmission and even made quite definitive estimates about the role of acute infections in overall HIV transmission [114]. Unfortunately, the methodology used for estimating this increased transmissibility completely ignores factors such as heterogeneity in susceptibility (see above). Proper estimation of the risk associated with acute infection per se would require a comparison between new, HIV-naïve partners of HIV-infected individuals in the acute and post-acute phase, which has never been done. Other, less understood, sources of heterogeneity in infectiousness also appear to exist. In a cross-sectional study from South Africa, viral loads varied by a factor of more than 10,000, but the factors underlying this heterogeneity are not completely clear [115]. Some factors have been recognized; for example, HLA-B57 and HLA-B27 genotypes have been associated with better viral control, and this leads one to expect that individuals with these HLA types are, on average, less infectious than others [116]. However, there is also within-person variation, and individuals may temporarily shed more infectious virions than usual [117]. Some anecdotal “superspreading" events suggest that heterogeneity in infectiousness can be substantial. A Zairian man in Belgium infected 11 out of 19 female sex partners, often after a single sexual contact [118]. Another case infected at least 11 of his many female sex partners [119]. It is unclear what this implies for HIV transmission in general, or the difference between transmission in Africa and other parts of the world. Africa has higher rates of tropical infections, including malaria, which may increase HIV viral load and transmission [120], [121]. Heterogeneity in infectiousness, as with heterogeneity in susceptibility, would explain why some exposed individuals seroconvert after a few sexual contacts, while others never acquire infection.

## Conclusions

We have identified several aspects of HIV transmission that may provide additional, alternative reasons for the rise and fall of HIV in high-prevalence countries in Africa. Current models may be misleading because factors such as heterogeneity in susceptibility, which may have substantially impacted the course of the HIV epidemic, have been ignored due to a lack of precise quantitative estimates. Key observations, such as non-linearity of the relationship between the number sex contacts and HIV transmission and the high risks associated with a seroconverting partner have consistently been interpreted, perhaps misinterpreted, in only a single way, while other explanations, with different epidemiological impacts, have been discounted. Thus, when incidence declines, the common assumption of fixed risk per sexual contact favors the conclusion of substantial changes in average sexual behavior.

What would our ideas imply for modeling? First, uncertainty in model structure, more than uncertainty in parameter values, dominates model uncertainty, and modelers should be aware of qualitatively different interpretations of available data. Micro-simulation can be used to effectively model complex, non-linear processes, in contrast to most models consisting of sets of differential equations. Modelers should explore alternative interpretations of available data and, together with epidemiologists and basic scientists, try to formulate empirical studies to discriminate between these interpretations. Scientific journals and reviewers, rather than encouraging conformity, should encourage and invite debate and dissenting interpretations of epidemiological studies. Understanding HIV's rise and fall could offer important lessons as this and future epidemics unravel.
